# Deep learning segmentation results in precise delineation of the putamen in multiple system atrophy

**DOI:** 10.1007/s00330-023-09665-2

**Published:** 2023-05-01

**Authors:** Alexander Rau, Nils Schröter, Michel Rijntjes, Fabian Bamberg, Wolfgang H. Jost, Maxim Zaitsev, Cornelius Weiller, Stephan Rau, Horst Urbach, Marco Reisert, Maximilian F. Russe

**Affiliations:** 1 Department of Neuroradiology, Medical Center – University of Freiburg, Faculty of Medicine, University of Freiburg, Freiburg, Germany; 2 Department of Diagnostic and Interventional Radiology, Medical Center – University of Freiburg, Faculty of Medicine, University of Freiburg, Freiburg, Germany; 3 Department of Neurology and Clinical Neuroscience, Medical Center – University of Freiburg, Faculty of Medicine, University of Freiburg, Freiburg, Germany; 4 Parkinson-Klinik Ortenau, 77709 Wolfach, Germany; 5 Medical Physics, Department of Diagnostic and Interventional Radiology, Medical Center, Faculty of Medicine, University of Freiburg, University of Freiburg, Freiburg, Germany; 6 Department of Stereotactic and Functional Neurosurgery, Medical Center - University of Freiburg, Faculty of Medicine, University of Freiburg, Freiburg, Germany

**Keywords:** Parkinsonian disorders, Putamen, Striatonigral degeneration, Multiple system atrophy, Deep learning

## Abstract

**Objectives:**

The precise segmentation of atrophic structures remains challenging in neurodegenerative diseases. We determined the performance of a Deep Neural Patchwork (DNP) in comparison to established segmentation algorithms regarding the ability to delineate the putamen in multiple system atrophy (MSA), Parkinson’s disease (PD), and healthy controls.

**Methods:**

We retrospectively included patients with MSA and PD as well as healthy controls. A DNP was trained on manual segmentations of the putamen as ground truth. For this, the cohort was randomly split into a training (*N* = 131) and test set (*N* = 120). The DNP’s performance was compared with putaminal segmentations as derived by Automatic Anatomic Labelling, Freesurfer and Fastsurfer. For validation, we assessed the diagnostic accuracy of the resulting segmentations in the delineation of MSA vs. PD and healthy controls.

**Results:**

A total of 251 subjects (61 patients with MSA, 158 patients with PD, and 32 healthy controls; mean age of 61.5 ± 8.8 years) were included. Compared to the dice-coefficient of the DNP (0.96), we noted significantly weaker performance for AAL3 (0.72; *p* < .001), Freesurfer (0.82; *p* < .001), and Fastsurfer (0.84, *p* < .001). This was corroborated by the superior diagnostic performance of MSA vs. PD and HC of the DNP (AUC 0.93) versus the AUC of 0.88 for AAL3 (*p* = 0.02), 0.86 for Freesurfer (*p* = 0.048), and 0.85 for Fastsurfer (*p* = 0.04).

**Conclusion:**

By utilization of a DNP, accurate segmentations of the putamen can be obtained even if substantial atrophy is present. This allows for more precise extraction of imaging parameters or shape features from the putamen in relevant patient cohorts.

**Clinical relevance statement:**

Deep learning-based segmentation of the putamen was superior to currently available algorithms and is beneficial for the diagnosis of multiple system atrophy.

**Key Points:**

• *A Deep Neural Patchwork precisely delineates the putamen and performs equal to human labeling in multiple system atrophy, even when pronounced putaminal volume loss is present.*

• *The Deep Neural Patchwork–based segmentation was more capable to differentiate between multiple system atrophy and Parkinson’s disease than the AAL3 atlas, Freesurfer, or Fastsurfer.*

**Supplementary Information:**

The online version contains supplementary material available at 10.1007/s00330-023-09665-2.

## Introduction

Multiple system atrophy (MSA) is an atypical Parkinson syndrome and therefore an important differential diagnosis in parkinsonism and especially Parkinson’s disease (PD). However, the initial clinical differentiation is difficult and misdiagnoses are rather common [1]. Pathophysiologically, MSA is an alpha-synucleinopathy, like PD, whereas in MSA further glial cytoplasmic inclusions occur in multiple regions of the brain [1].

Multiple studies have evaluated MRI-based parameters to achieve a deeper understanding of the pathophysiology and pathogenesis of MSA in vivo [2–8]. The exact segmentation of target structures is essential to read out sequence parameters, e.g. from different diffusion models, or to perform radiomic analyses, e.g. using shape features. A commonly used approach is to extract sequence parameters in a region-of-interest (ROI) approach. Standardized brain atlases or Freesurfer-derived segmentations are routinely employed to delineate pathognomonic regions. However, in diseases with pronounced regional atrophy, these techniques might be limited by insufficient precision, leading to a potential bias in the extraction of sequence parameters due to inaccurate segmentation are conceivable.

Manual segmentation of brain structures, while still an accurate method especially in atrophy, is time-consuming and potentially interreader variable [9]. In contrast to Freesurfer and probabilistic approaches, artificial intelligence techniques such as deep learning are more capable of detecting atrophic structures [10]. Therefore, Fastsurfer was recently introduced as a Freesurfer-based technique that uses a deep learning approach to improve the speed of analysis and is better suited for dementia-associated atrophy [10]. In addition, the improved brain structure segmentation of a deep learning approach might also be of diagnostic value, since the discriminative potential of volumetry has been demonstrated in atypical Parkinson syndromes [11, 12].

Deep learning–based image segmentations frequently rely on the U-Net or variations of a U-shaped network. These algorithms are based on convolutional neural networks that are also commonly used for biomedical image segmentation. The network is composed of a contracting path and an expanding path, which allows it to effectively learn features and therefore segmentations from images [13]. As an example of automatic segmentation of a complex anatomical brain structure, the claustrum was successfully segmented using a multiview 2D convolutional neural network architecture based on a U‐shape network [14].

Approaches to using a full high-resolution 3D image sample are often limited by available hardware resources, particularly the limited memory of the graphics processing units used for network training. A typical technique to solve this limitation is to break the task into smaller subtasks by dividing the image data into smaller image patches. However, this often leads to a loss of global context, which could limit the effectiveness of these approaches. Deep Neural Patchworks (DNP) is a segmentation framework that is based on hierarchical and nested stacking of patch-based 3D networks of fixed matrix size but decreasing physical input size, which solves the dilemma between global context and memory limitations in high-resolution 3D images [15]. This framework has shown its potential for segmenting small image structures of head CT scans for computer-assisted craniomaxillofacial surgery with high accuracy [16].

Therefore, the aim of this study was to develop a Deep Neural Patchwork for the segmentation of the putamen in MSA and PD. Also, we compared the accuracy of the deep neural patchwork segmentation with an established brain atlas, Freesurfer, and deep learning accelerated Fastsurfer and evaluated the diagnostic value of volumetric parameters based on deep neural patchwork segmentation.

## Materials and methods

### Subjects

This retrospective bicentric study included patients who underwent MRI for the differential diagnosis of neurodegenerative Parkinson syndromes or the evaluation of advanced treatment options between 01/2018 and 12/2021. Patients in whom MSA or PD was either clinically established or probable were recruited. Clinical diagnoses were validated by two experts in movement disorders (M.R., board-certified neurologist; W.H.J., board-certified neurologist; N.S., > 7 years of neurology training) based on current diagnostic criteria; all available medical records were used for the best clinical lifetime diagnosis [17, 18].

We included age and sex-matched healthy controls (HC) as a comparison group. Subjects in the HC group had no known neurological medical condition, no neurological deficits on clinical examination, and no family history of neurodegenerative Parkinson syndromes. Furthermore, this group of subjects did not show neuropsychological impairment according to the Montreal Cognitive Assessment [19].

The study was approved by the Institutional Review Board (Ethics Committee – University of Freiburg; EK 22/1270) and carried out in accordance with the Declaration of Helsinki and its later amendments. Due to the retrospective nature of this study, the need for written informed consent was waived.

### MRI examination protocol

MRI was performed with a 3-Tesla scanner (MAGNETOM Prisma, Siemens Healthcare) with a 64-channel head and neck coil. Patients with Parkinson syndromes were in the ON-state during acquisition. T1‐weighted (T1w) images were acquired with a three‐dimensional (3D) magnetization‐prepared 180° radio frequency pulses and rapid gradient‐echo (MPRAGE) sequence (repetition time: 2500 ms, echo time: 2.82 ms, flip angle: 7°, TI = 1100 ms, GRAPPA factor = 2, 1.0 mm^3^ isotropic voxels, 192 contiguous sagittal slices). In addition, 3D T2w FLAIR and 2D SWI data were obtained as given in Supplementary Table 1.

### AAL3-, Freesurfer-, and Fastsurfer-based putamen segmentation

All data processing was implemented within our in-house post-processing platform (NORA; www.nora-imaging.com). T1w imaging datasets were automatically segmented into white and gray matter and spatially normalized using CAT12 (http://www.neuro.uni-jena.de/cat/). From this, the AAL3 atlas was employed to create the ROI of the putamen [20].

We obtained an automatic segmentation of the putamen from the FreeSurfer toolbox [21] Version 6.0 and employed the recently introduced Fastsurfer approach [10] after parcellation according to the Desikan-Killiany atlas (DKT) [22].

### Deep Neural Patchworks for putamen segmentation

**Fig. 1:**
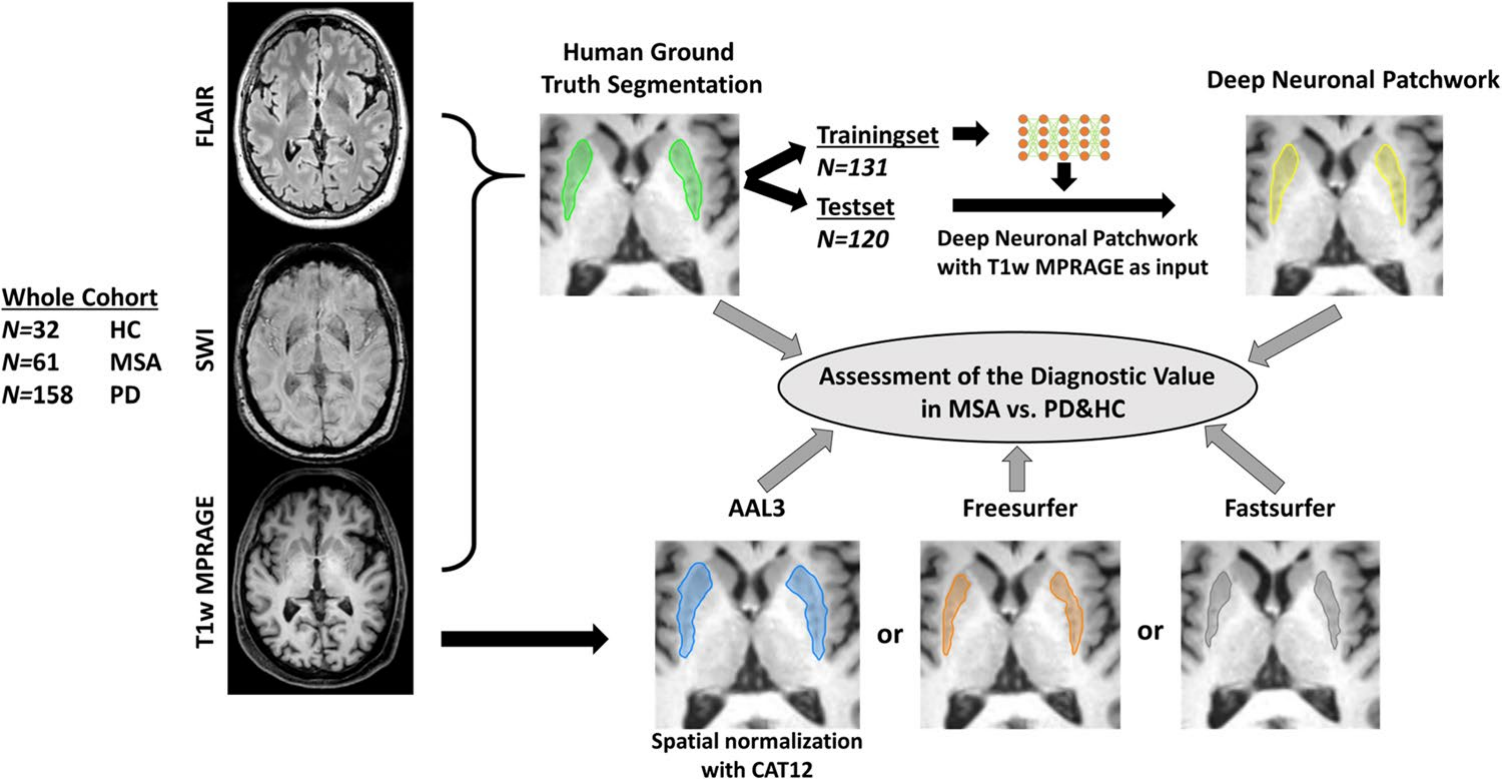
Schematic of the workflow of the image data labeling and training of the Deep Neural Patchwork. *HC* healthy controls; *MSA* multiple system atrophy; *PD* Parkinson’s disease; *FLAIR* fluid-attenuated inversion recovery; *SWI* susceptibility weighted imaging; *MPRAGE* magnetization prepared rapid gradient echo

Left and right putamen were manually segmented on 3D reformatted T1w MPRAGE images under simultaneous consideration of the 3D FLAIR and SWI by an experienced neuroradiologist (A.R., 5 years of experience in neuroimaging) blinded to clinical diagnosis as depicted in Fig. 1 to serve as ground truth for further processing and analyses. Primary segmentation of the putaminal outline was manually done on axial slices and correction of the outline was performed on coronal and sagittal reformats. This manual segmentation served as ground truth for further DNP training and testing of the different methods. The data leakage was omitted due to the patient-level separation of training and test data. A single case per class was randomly drawn from the training sample for monitoring the training process as validation cases. DNP was used as a segmentation framework that is based on hierarchical and nested stacking of patch-based 3D networks of fixed matrix size but decreasing physical input size. The size of the hierarchical pyramid was selected such that it allowed for a reasonable 3D field-of-view of each dimension of 150 mm in the coarsest layer and a very high spatial resolution in the smallest layer with an isotropic resolution of 1 mm. The matrix size of 32^3^ voxels was selected in a way that would map representative portions of the anatomy. These settings were limited by the available hardware capacity for sample size and pyramid size. The architecture of the basis U-Net applied in this project is close to the default U-Net configuration with feature dimensions (8, 16, 16, 32, 64) and maximum pooling in the encoding layers and transposed convolutions in the decoding layers. The network is trained with the Adam optimizer with a learning rate of 0.001. As a loss function, a binary cross-entropy variant of the top-K loss was used. The Patchwork CNN was trained for 5 million patches. The training took around 20 h with a batch size of 150 images in the graphic unit ‘s memory. Training was performed on a GPU-accelerated server system using an RTX A6000 graphic unit (NVIDIA). During training, patches were randomly sampled so that approximately 80% of the finest patches contained at least one label. No systematic tuning was done with the settings adapted to prior established values [16]. To assess the performance of the hierarchical structure of the DNP, we trained a more classical single-level 3D U-Net with a matrix size of 128^3^, consequently reducing the spatial resolution to 2×2×1.5 mm [23]. The batch size had to be limited to 10 samples due to the high hardware demands of such a large 3D U-Net.

To create the results in the separate test dataset, a random patching scheme was used, combined with a tree-like branching sampling method. In detail, six nested patches were randomly drawn from the coarsest level, and the three patches with the highest probability of containing a label were selected for further analysis at the next nested level. This process was repeated overall levels, allowing for efficient coverage of relevant image parts. The prediction of a full volume was performed on a 16-core CPU machine (without a GPU), which, corresponding to the used image volume, took around 2 min.

### Statistics

Statistical analyses were performed using R (version 4.1.0, https://www.R-project.org/). Data are presented as the mean and standard deviation for continuous variables and as absolute frequencies and percentages for categorical variables. The Shapiro–Wilk test was used to assess the normal distribution of data. Group differences were assessed by the chi-squared test and Mann–Whitney-U test. Intergroup differences were assessed through analysis of variance (ANOVA), followed by Tukey’s honest significance test. The performance of the AAL3 atlas, the Freesurfer- and Fastsurfer-approach, and DNP in comparison to the human labeling was assessed via the dice coefficient and the 95% Hausdorff distance. We plotted the ROC curves of the putaminal volume for the differential diagnosis of MSA vs. PD and HC within the test cohort and the AUC and cut-off values were calculated. DeLong’s test was employed to compare the AUCs. We additionally assessed the respective algorithm´s performance in Bland–Altman plots. The significance threshold was set to *p* < 0.05.

### Data and code availability

Data are available from the authors upon reasonable request and approval of the local ethics committee. Code of the DNP Framework *and a standalone version for putamen segmentation are* available on bitbucket.org/reisert/patchwork.

## Results

### Subjects

We report on 32 HC, 61 patients with MSA, and 158 patients with PD. There were no group differences in terms of age (*p* = 0.49) between the HC, MSA, or PD groups, whereas more male patients were included in the PD group than in the HC or MSA group (*p* < 0.001). The disease duration in the PD group was significantly longer than in the MSA group (*p* < 0.001). Further patient characteristics are provided in Table 1.

**Table 1:** Participant characteristics

	Healthy controls (*N* = 32)	Multiple system atrophy (*N* = 61)	Parkinson’s disease (*N* = 158)
Sex			
F	18 (56.3%)	36 (50.0%)	50 (31.6%)
Age (years)			
Mean (SD)	63.4 (9.3)	65 (8.9)	65.5 (8.7)
UPDRS-III in OFF-state			
Mean (SD)	NA (NA)	48.1 (18.7)	46.7 (18.3)
Disease duration (years)			
Mean (SD)	NA (NA)	3.8 (3.6)	9.5 (5.7)

Prior to DNP training, we randomly split our cohort into a training set (*N* = 131: 17HC, 30MSA, 84PD) and a test set (*N* = 120: 15HC, 31MSA, 74PD) that did not differ in terms of age and sex (*p* > 0.05).

### Performance of the algorithms for putamen segmentation

**Fig. 2:**
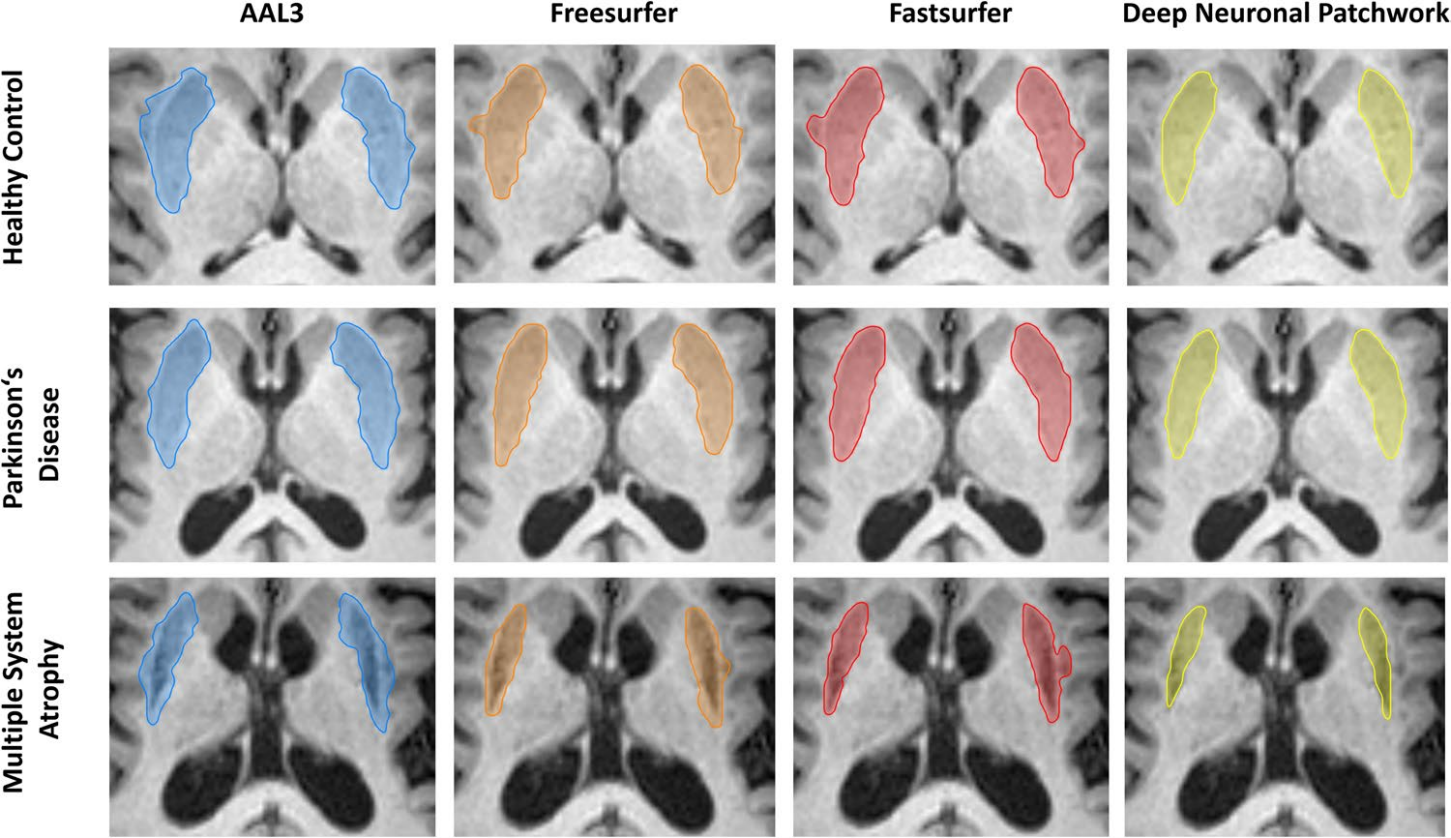
Exemplary segmentation outputs of Automatic anatomical labeling 3 (AAL3), Freesurfer, Fastsurfer, and the Deep Neural Patchwork in a healthy control (66 years old female), a patient with Parkinson’s disease (49 years old female) and a patient with multiple system atrophy (68 years old female)

Human ground truth labeling resulted in significantly lower putaminal volume in MSA (2.62 ± 1.03 mL) compared to HC (4.61 ± 0.54 mL; *p* < 0.001) and PD (4.34 ± 0.53 mL; *p* < 0.001), while no difference between HC and PD was found (*p* = 0.36). We successfully obtained putaminal segmentations from all employed algorithms, i.e. AAL3, Freesurfer, Fastsurfer, and DNP as depicted in Fig. 2, and observed no side differences within the respective algorithm outputs (all *p* > 0.05, please see Supplementary Fig. 1).

**Fig. 3:**
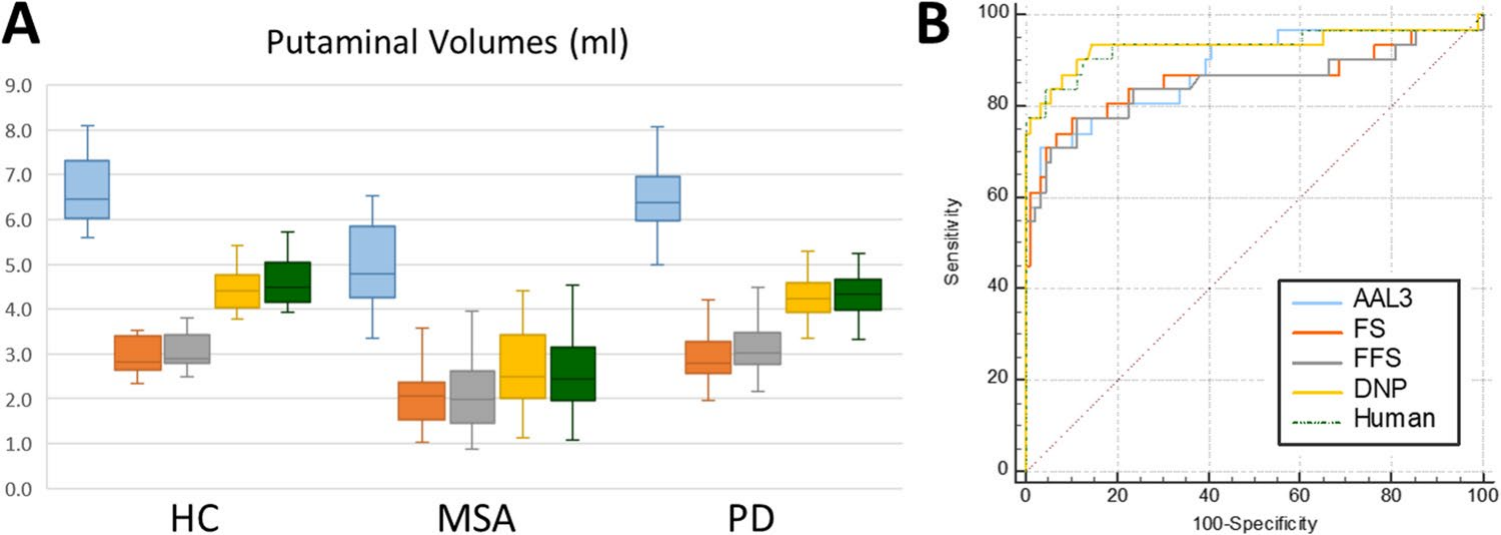
**A** Mean putaminal volumes (mL) of the different segmentations and (**B**) receiver operating characteristics for the identification of patients with multiple system atrophy vs. patients with Parkinson’s disease and healthy controls. In this, the Deep Neural Patchwork (DNP) performed superiorly compared to the AAL3 atlas and the Freesurfer (FS) and Fastsurfer (FFS) approaches (*p* < 0.05). AAL3-related data are given in blue, Freesurfer in red, Fastsurfer in gray, DNP in yellow, and human labeling in green

All algorithms revealed significantly lower putaminal volume in MSA in comparison to HC and PD, too, as given in Fig. 3. Regarding the mean putaminal volume within all groups, compared to human ground truth labeling (3.93 ± 1.04 mL), the AAL3 output resulted in a structurally larger volume (6.17 ± 1.04 mL; *p* < 0.001), whereas Freesurfer and Fastsurfer resulted in structurally smaller volumes (2.81 ± 0.85 mL and 2.98 ± 0.94, respectively; both *p* < 0.001). Good agreement was found between the ground truth and the DNP (3.88 ± 0.96 mL; *p* = 0.99).

This is corroborated by a significantly lower dice coefficient of 0.72 ± 0.08 for AAL3 (*p* < 0.001), 0.82 ± 0.06 for Freesurfer (*p* < 0.001), and 0.84 ± 0.05 for Fastsurfer (*p* < 0.001), compared to 0.96 ± 0.02 for the DNP as well as better 95% Hausdorff distance of the DNP (0.89 ± 0.30) compared to AAL3 (3.76 ± 1.28; *p* < 0.001), Freesurfer (2.79 ± 1.16; *p* < 0.001), and Fastsurfer (2.76 ± 1.16; *p* < 0.001). The more classical single-level 3D-Unet also performed inferior to the DNP, as it reached dice-coefficients of 0.90 ± 0.04 (*p* < 0.001).

A more detailed analysis after the separation of the diagnoses revealed that AAL3, Freesurfer, Fastsurfer, and the DNP had lower dice scores in MSA compared to HC or PD (all *p* < 0.001). More details are presented in Table 2. The dice coefficient results are corroborated by the Bland–Altman plots (please see Supplementary Fig. 1).

**Table 2:** Dice and 95% Hausdorff’s distance split and compared among groups

	Dice (mean ± SD)			95% Hausdorff’s distance (mean ± SD)		
	HC	MSA	PD	HC	MSA	PD
AAL3	0.78 ± 0.02	0.63 ± 0.09	0.75 ± 0.04	2.78 ± 0.37	4.86 ± 1.19	3.43 ± 0.95
Freesurfer	0.86 ± 0.02	0.76 ± 0.06	0.84 ± 0.04	1.79 ± 0.41	3.84 ± 1.08	2.55 ± 0.98
Fastsurfer	0.88 ± 0.01	0.78 ± 0.06	0.86 ± 0.04	1.78 ± 0.48	3.61 ± 1.01	2.60 ± 1.09
DNP	0.97 ± 0.01	0.93 ± 0.03	0.96 ± 0.01	0.80 ± 0.25	1.03 ± 0.19	0.84 ± 0.33
	HC vs. MSA	HC vs. PD	MSA vs. PD	HC vs. MSA	HC vs. PD	MSA vs. PD
AAL3	< .001	0.26	< .001	< .001	0.06	< .001
Freesurfer	< .001	0.12	< .001	< .001	0.02	< .001
Fastsurfer	< .001	0.18	< .001	< .001	0.02	< .001
DNP	< .001	0.54	< .001	0.03	0.88	0.01

Intergroup differences were assessed through analysis of variance (ANOVA), followed by Tukey’s honest significance test. *AAL3* automatic anatomic labelling; *DNP* Deep Neural Patchwork; *HC* healthy controls; *MSA* multiple system atrophy; *PD* Parkinson’s disease

### Comparison of diagnostic value

To access the diagnostic value of the respective segmentations, we calculated the AUC-ROC for the comparison of MSA vs. PD and HC. The AAL3-derived segmentation revealed an AUC of 0.88 (95% CI 0.81–0.94; cut point 5.51 mL), 0.86 for Freesurfer (95% CI 0.79–0.92; cut point 2.33 mL), 0.85 for Fastsurfer (95% CI 0.78–0.91; cut point 2.61 mL), and 0.93 for the DNP (95% CI 0.87–0.97; cut point 3.77 mL), whereas the human ground truth labeling’s AUC was 0.93 (95% CI 0.87–0.97; cut point 3.57 mL). DeLong’s test revealed that the AUC of the DNP was superior to AAL3 (*p* = 0.02), Freesurfer (*p* = 0.048), and Fastsurfer (*p* = 0.04).

## Discussion

Using a well-characterized cohort of patients with MSA, PD, and healthy controls, we trained a DNP to validly segment the putamen. In doing so, the DNP outperformed the established AAL3 atlas and Freesurfer as well as Fastsurfer. This was reflected in close to equal volumes compared to expert manual segmentation and in the higher diagnostic accuracy of DNP-based segmentation in identifying MSA patients.

In general, our ground truth labels matched the range of putamen volume in HC, MSA, and PD of previous studies [12, 24–29]. In comparison with ground truth labels, we observed that the AAL3 algorithm tended to overestimate the volume of the putamen, whereas Freesurfer and Fastsurfer tended to underestimate it. In contrast, the DNP output was close to human labeling. The dice-coefficient of the Freesurfer output was within the same range as a study that related the segmentation of the putamen in thirty healthy probands between two human labelers (dice-coefficient human vs. human 0.9) with the output of Freesurfer (dice-coefficient of human vs. different Freesurfer versions: 0.82–0.9) [24].

Of note, all employed techniques had performed worse delineating the putamen in the MSA group than in HC and PD, though the difference in dice scores was substantially smaller in the DNP approach. Therefore, the major challenge for the approaches based on AAL3, Freesurfer, and Fastsurfer was the insufficient identification of putaminal atrophy in MSA, reflected on the one hand by the low dice-coefficient and the worse 95% Hausdorff distance compared to DNP, but also by the smaller AUCs. This is mainly because neither AAL3 nor the Freesurfer nor the Fastsurfer approach used patients with putaminal atrophy for labeling and therefore lack this pathological spectrum. An MSA-specific brain atlas seems rather inconvenient in the clinical setting since atrophy of the putamen is variable within the disease spectrum [12]. In contrast, the DNP was sensitive to a variable range of pathological changes in the sense of putaminal atrophy due to the pixel-based segmentation approach and successfully applied to patients with MSA and additionally to patients with PD as an important differential diagnosis. Of note, the hierarchical patch-based 3D architecture of the employed DNP outperformed the Fastsurfer approach which itself relies on deep learning techniques in the sense of a combination of 2D networks that assess image data as multislice input in different reformats. It also outperformed a classical single-level 3D U-Net with a high matrix size trained on the identical dataset with comparable training time and hardware resources.

In principle, a fair diagnostic value of the volumetry of the putamen is also given for AAL3, Freesurfer, and Fastsurfer, since the volumes were under- respectively overestimated in a structural manner. However, these approaches are insufficient for more in-depth analyses. Incorrect segmentations do not allow for accurate radiomic-based shape analyses. Such assessment of shape features might corroborate the known non-uniform affection of neurodegeneration within the putamen in MSA with dorsolateral accentuation [30]. In addition, incorrect segmentation can significantly influence the utilization of quantitative image parameters, which are frequently employed to assess neurodegenerative processes. In this respect, accurate segmentation is essential to identify the association between clinical symptoms and central nervous system alterations in neurodegenerative diseases as such an approach yields great potential in both diagnostic approaches and in the study of the pathophysiology in vivo [31, 32].

Our DNP approach can identify the putamina in a side-separated manner, this is particularly important for PD, as it is a commonly clinically lateralizing disease, and asymmetries are also known in functional imaging, whereas controversial results are reported for MRI [32–35].

The fact that the image data were collected only in a bicentric setting with a uniform examination protocol limits the generalizability of the model. However, it has been shown that segmentation algorithms based on structural non-contrast-enhanced 3D T1w image data are validly translatable [36]. Although segmentation was performed blinded to diagnosis, there is a potential bias in that SWI was available for segmentation, and signal extinctions at the lateral edge of the putamen are known to occur here in patients with MSA [37].

In conclusion, a DNP allows for accurate segmentation of the putamen even if substantial atrophy is present. Precise delineation of the putamen enables the extraction of imaging parameters or analyses of shape features.

### Supplementary Information

Below is the link to the electronic supplementary material.

Supplementary file1 (PDF 267 kb)

## Abbreviations

DNP: Deep Neural Patchworks
HC: Healthy controls
MSA: Multiple system atrophy
PD: Parkinson’s disease
