# Supplementary material for: Deep learning segmentation results in precise delineation of the putamen in multiple system atrophy
Source: Eur Radiol. 2023 May 1;33(10):7160–7. doi: 10.1007/s00330-023-09665-2 (PMC10511621; doi:10.1007/s00330-023-09665-2)
Supplement: Supplementary file 1 — Supplementary file1 (PDF 267 kb) [file 330_2023_9665_MOESM1_ESM.pdf]

Supplement

Supplementary Table 1 Neurodegeneration-dedicated MRI protocol (3-Tesla MAGNETOM Prisma, Siemens Healthcare, Erlangen, Germany).

MRI sequence	No. of slices/ thickness (mm)	Voxel size (mm^3)	TI/TR/TE/α (ms/ms/ms/°)	acquisition time (min:sec)
**Sagittal 3D MPRAGE**	160/1	1x1x1	1100/2500/2.82/7	3:58
**Sagittal 3D FLAIR-SPACE**	160/1	1x1x1	1800/5000/388/var	6:52
**Axial 2D SWI**	72/2	0.9x09x2	-/27/20/17	2:39

MPRAGE Magnetization Prepared Rapid Gradient Echo, FLAIR SPACE Fluid-Attenuated Inversion Recovery - sampling perfection with application-optimized contrasts by using flip angle evolution, TSE Turbo Spin Echo, TI inversion time, TR repetition time, TE echo time, α flip angle

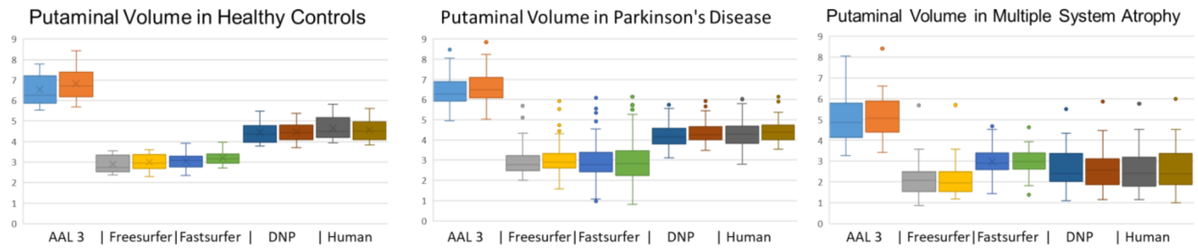

Supplementary Figure 1 Putaminal volumes (ml) for both sides (left segmentation is given as the left boxplot for the employed approach respectively) as obtained by the different outputs and split by the groups

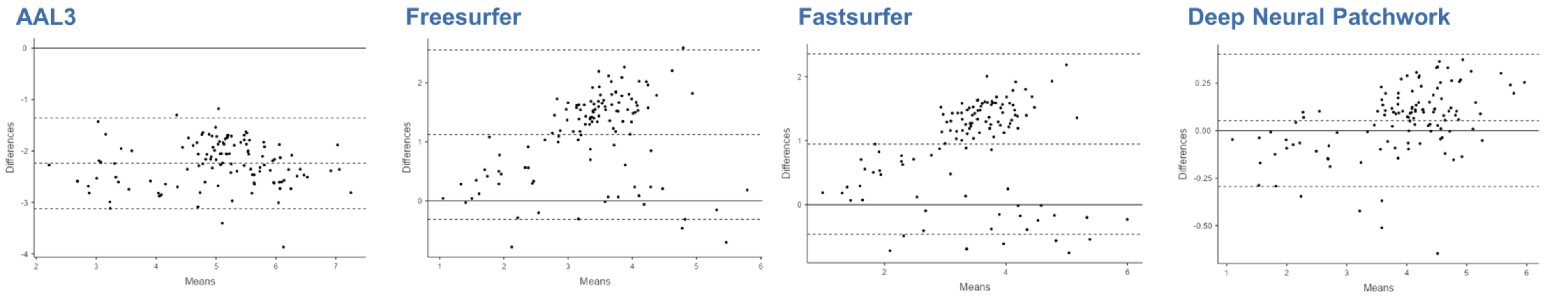

AAL3

	Estimate	95% Confidence Interval Lower	Upper
Bias (n = 120)	-2.24	-2.32	-2.16
Lower limit of agreement	-3.12	-3257	-2.98
Upper limit of agreement	-1.36	-1.49	-1.22

Freesurfer

	Estimate	95% Confidence Interval Lower	Upper
Bias (n = 120)	1.12	0.99	1.26
Lower limit of agreement	-0.31	-0.54	-0.09
Upper limit of agreement	2.56	2.33	2.79

Fastsurfer

	Estimate	95% Confidence Interval Lower	Upper
Bias (n = 120)	0.95	0.82	1.08
Lower limit of agreement	-0.46	-0.68	-0.24
Upper limit of agreement	2.35	2.13	2.58

Deep Neural Patchwork

	Estimate	95% Confidence Interval Lower	Upper
Bias (n = 120)	0.05	0.02	0.08
Lower limit of agreement	-0.29	-0.35	-0.24
Upper limit of agreement	0.40	0.35	0.46

Supplementary Figure 1 Bland-Altman plots showing the mean difference (ml) between the respective algorithm vs. human ground truth labeling and the overall average versus the overall average regarding the putaminal volume.
